# Racial disparities in infant mortality: what has birth weight got to do with it and how large is it?

**DOI:** 10.1186/1471-2393-10-86

**Published:** 2010-12-28

**Authors:** Timothy B Gage, Fu Fang, Erin K O'Neill, A Gregory DiRienzo

**Affiliations:** 1 Department of Anthropology, University at Albany, State University of New York, 1400 Washington Ave., Albany, NY 12222, USA; 2 Department of Epidemiology and Biostatistics, University at Albany, State University of New York, 1400 Washington Ave., Albany, NY 12222, USA

## Abstract

**Background:**

It has been hypothesized that birth weight is not on the causal pathway to infant mortality, at least among "normal" births (i.e. those located in the central part of the birth weight distribution), and that US racial disparities (African American versus European American) may be underestimated. Here these hypotheses are tested by examining the role of birth weight on racial disparities in infant mortality.

**Methods:**

A two-component Covariate Density Defined mixture of logistic regressions model is used to decompose racial disparities, 1) into disparities due to "normal" versus "compromised" components of the birth cohort, and 2) further decompose these components into indirect effects, which are associated with birth weight, versus direct effects, which are independent of birth weight.

**Results:**

The results indicate that a direct effect is responsible for the racial disparity in mortality among "normal" births. No indirect effect of birth weight is observed despite significant disparities in birth weight. Among "compromised" births, an indirect effect is responsible for the disparity, which is consistent with disparities in birth weight. However, there is also a direct effect among "compromised" births that reduces the racial disparity in mortality. This direct effect is responsible for the "pediatric paradox" and maybe due to differential fetal loss. Model-based adjustment for this effect indicates that racial disparities corrected for fetal loss could be as high as 3 or 4 fold. This estimate is higher than the observed racial disparities in infant mortality (2.1 for both sexes).

**Conclusions:**

The results support the hypothesis that birth weight is not on the causal pathway to infant mortality among "normal" births, although birth weight could play a role among "compromised" births. The overall size of the US racial disparities in infant mortality maybe considerably underestimated in the observed data possibly due to racial disparities in fetal loss.

## Background

It has been argued that birth weight may not be on the "causal pathway" to infant mortality [1-3]. The best developed argument, originating with the Wilcox-Russell hypothesis [2,4,5], is supported by qualitative analyses using directed acyclic graphs [6]. Both of these approaches are based on simple graphical observations of the response of birth weight and birth weight specific infant mortality to exogenous stressors, such as smoking or altitude. The Wilcox-Russell hypothesis [2,4,5] suggests that in response to a stressor, the birth weight specific infant mortality curve and birth weight distribution appear to shift right or left together resulting in no change in total mortality. Consequently, there is no indirect effect of the stressor due to the shift in birth weight. Any changes in mortality due to the stressor are hypothesized to be due to the entire mortality curve shifting up or down independently of birth weight, i.e. direct effects of the stressor. Complementary analyses using directed acyclic graphs identify three plausible models which could account for the dynamics of birth weight specific mortality [6]. One model supports the Wilcox-Russell hypothesis [2,4,5], while the other two include birth weight on the "causal pathway" [6].

**Figure 1:**
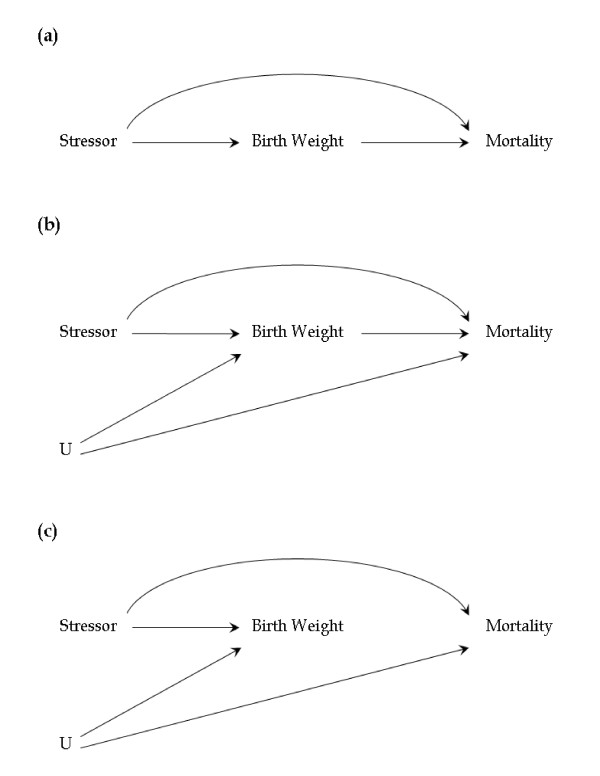
**Three directed acyclic graphs considered to be plausible models of the relationship of birth weight to infant mortality in response to a stressor (adapted from **[6]**)**. Model (a) assumes that the stressor has direct effects on birth weight and mortality, birth weight has direct effects on infant mortality, and an interaction of the stressor and birth weight are assumed to account for the reverse-J-shaped birth weight specific mortality curve. Model (b) also assumes that the stressor has direct effects on birth weight and mortality, birth weight has direct effects on infant mortality, and unobserved covariates U account for the reverse-J shape. Model (c) assumes that the stressor has direct effects on birth weight and mortality, the reverse-J shape is the result of unobserved covariates U, but birth weight does not have direct effects on mortality.

The three plausible directed acyclic graphs are illustrated in Figure 1. Figure 1a assumes that the stressor has direct effects on birth weight and mortality and birth weight has direct effects on infant mortality. In this case, an interaction between the stressor and birth weight is assumed to be responsible for the reverse-J shape of the birth weight specific infant mortality curve. Hernández-Diaz et al. [6] consider this the least likely model since the interaction would need to be complex. Figure 1b also assumes that the stressor has direct effects on birth weight and mortality and birth weight has direct effects on infant mortality. In this case, unobserved covariates U are assumed to account for the reverse-J-shaped birth weight specific mortality. Although an interaction between the stressor and birth weight could also contribute to the reverse-J-shaped curve. Finally, Figure 1c assumes that the stressor has direct effects on birth weight and mortality, but birth weight does not have direct effects on mortality. In this case, the reverse-J shape is entirely the result of unobserved covariates U. Figure 1c corresponds to the Wilcox-Russell hypothesis [2,4,5]. The direct effect of the stressor on mortality is responsible for a simple increase or decrease in mortality independent of birth weight. The direct effect of the stressor on birth weight is responsible for a shift right or left in the birth weight density, and the mortality curve shifts with it because birth weight does not affect mortality. In Figures 1a and 1b, birth weight does affect mortality and hence the birth weight density and birth weight specific mortality curve are not coupled. This uncoupling could be due to a simple differential shift in the birth weight density and birth weight specific infant mortality curve as assumed in the Wilcox-Russell hypothesis [2,4,5]. Or, as suggested by Hernández-Diaz et al. [6], it could also be due to a change in the shape of the birth weight specific mortality curve due to an interaction of the stressor and birth weight on infant mortality. Wilcox and Russell [2,4,5] assume the shape of the birth weight specific mortality curve is constant.

If birth weight is not on the "causal pathway", i.e. it does not mediate the effect of race on infant mortality (Figure 1c), then the US national policy of reducing infant mortality [7] in general, and racial disparities in particular, by reducing the low birth weight rate might not be effective. On the other hand, the Wilcox-Russell hypothesis [2,4,5] only applies to "normal" births (which they defined as those in the central part of the birth weight distribution) and not all births [5], so birth weight could still mediate the effect of race on infant mortality among the remaining births. An initial quantitative statistical test of the Wilcox-Russell [2,4,5] and Hernández-Diaz et al. [6] hypotheses using Covariate Density Defined mixture of logistic regressions (CDDmlr) with maternal age as a stressor supports the argument that birth weight is not on the causal pathway to infant mortality for either "normal" or the remaining "compromised" births [8].

Wilcox and others [5,9-11] have also argued that racial disparities in infant mortality may be underestimated. This view is based on the simple graphical observation that lower birth weight African American births have better survival than their European American peers with similar birth weight despite much higher mortality overall, i.e. the racial "birth weight or pediatric paradox". The hypothesis is that unmeasured (and hence uncontrolled) heterogeneity between the racial groups might mask part of the true racial disparities. It has been shown that CDDmlr isolates the race "pediatric paradox" within the "compromised" subpopulation, allowing better control of this phenomenon [10].

The objective of this paper is to quantitatively document the role that birth weight plays in racial disparities in infant mortality using the 2001 United States non-Hispanic African and European American birth cohorts controlling for sex. In particular, we statistically test the hypothesis that birth weight is on the "causal pathway" to infant mortality and decompose racial disparities in infant mortality into effects, which are independent of birth weight (direct effects of race) and effects, which are due to the racial disparities in birth weight (indirect effects of race mediated by birth weight). A secondary aim is to estimate the magnitude of the racial disparities in infant mortality while controlling for the "pediatric paradox". We do not propose that "race" is the cause of these disparities, but simply a proxy for a collection of stressors (e.g. socio-economic status, education, and genetic etc, some of which may be unobserved), which are the underlying causes of these differences.

## Methods

### Data Source

The data for this analysis are obtained from 2001 NCHS Birth Cohort Linked Birth/Infant Death data set. Race and ethnic origin are based on mother's reported race and ethnic origin. Approximately 6.4% and 8.7% of the non-Hispanic European and non-Hispanic African American births, respectively, are excluded from this analysis due to missing information or LMP gestational age <20 weeks or birth weight <500 grams. Summary statistics for the samples used are presented in Table 1. These data are public use samples, freely distributed by NCHS and used with permission.

**Table 1:** Descriptive statistics for the 2001 sample populations

Birth Cohort	# Births	# Deaths	CDR	Birth Weight (grams)			
				
				Min	Mean	Median	Max
Eur. Am. F.	1,023,583	3,558	3.48	500	3342	3365	6350
Eur. Am. M.	1,076,814	4,880	4.53	500	3461	3487	7858
Af. Am. F.	255,758	1,865	7.29	500	3092	3135	7002
Af. Am. M.	264,130	2,545	9.64	500	3200	3260	7220

Eur. = European; Af. = African; Am. = American; F. = females; M. = males

CDR = Crude death rate (death per 1000 births)

### Statistical Model - CDDmlr

Covariate Density Defined mixture of logistic regressions (CDDmlr), while a generally applicable statistical procedure, was specifically designed to test the Wilcox-Russell hypothesis [8]. It decomposes the birth weight distribution into a number of subpopulations, using standard mixture of Gaussian distributions, and simultaneously fits a separate birth weight specific mortality curve to each of the subpopulations identified by the birth weight density submodel [10]. A two-component CDDmlr model using Gaussian distributions (truncated at 500 grams) and logistic regressions (a 2^nd ^degree polynomial of birth weight) is the parsimonious model, that fits birth weight distributions [12] and birth weight specific mortality curves [9,10] remarkably well. One subpopulation accounts for most births in the center of the birth weight distribution and appears to identify "normal" births, while the other accounts for most low and macrosomic births, and is hence called "compromised" births [9,10,12]. Clearly the "compromised" subpopulation represents a heterogeneous group, i.e. births "compromised" by a variety of potential factors. However, increasing the number of subpopulations does not resolve the "compromised" subpopulation into separate groups [13] and placing constraints on the fitting process [4] simply reduces the goodness of fit. The model represents the maximum likelihood division of the birth weight distribution given the assumption that the birth weight distribution is the sum of two Gaussian subpopulations. Furthermore, the "compromised" subpopulation differs slightly from Wilcox's "residual" subpopulation [4] given that it also accounts for births in the normal birth weight range, where as Wilcox's "residual" births [4] were restricted to the lower tail. However, a number of clinicians have argued that "compromised" births do occur in the normal birth weight range, but are not recognized as "compromised" when using the arbitrary low birth weight standards (i.e. <2500 grams) and are hence understudied [14,15]. Given that the Reverse-J-shaped birth weight specific mortality curves fitted to each of the two subpopulations (i.e. "normal" and "compromised") is parsimonious [9,10], we assume that the reverse-J shape is due to other unspecified covariates and not a "causal" effect of birth weight. This is consistent with Hernández-Diaz and her colleagues' assumption [6] that the reverse-J shape of the mortality curve is due to other unmeasured covariates, such as the theory of Basso and Wilcox [16,17] that the reverse-J shape is due to confounding. Here, we use CDDmlr to statistically examine the Wilcox-Russell [2,4,5] and Hernández-Diaz et al. [6] hypotheses for both "normal" and "compromised" births. In addition, since the "pediatric paradox" is associated with the "compromised" subpopulation [10], CDDmlr can control for this phenomenon as well.

The model employed here is an extension of the two-subpopulation birth weight only CDDmlr model of infant mortality [10]. In brief, a stratified CDDmlr model is constructed by defining the five parameters (referred to collectively as *θ*) in the birth weight density submodel and the six parameters (referred to collectively as *β *or β*, representing the two 2^nd ^degree polynomials of birth weight or standardized birth weight, respectively) in the mortality submodel of the basic CDDmlr model [10] as linear functions of a dummy variable (e.g. race). Thus this stratified model can quantify the differences in the birth weight distribution (i.e. the proportion of "compromised" births, and the means and standard deviations of both subpopulations) and the (standardized) birth weight specific mortality characteristics between African and European American birth cohorts. In this study, birth weight is standardized (Z-scored) for each subpopulation based on the subpopulation specific mean and variance. This step essentially breaks the association of race and birth weight so that we can estimate the birth weight independent effect (direct effect) and any remaining birth weight dependent effect [18,19]. The latter may be potentially due to a direct effect of birth weight on infant mortality, or uncontrolled confounding between birth weight and infant mortality, or an interaction of race and birth weight on infant mortality. In particular, we investigate the effects of race on:

(i) the logit of minimum mortality (i.e. a vertical shift of the mortality curve by race, the direct effect of race);

(ii) the optimal standardized birth weight (i.e. a horizontal shift of the mortality curve by race, the indirect effect of race described by Wilcox-Russell [2,4,5]); and

(iii) the particular shape of the reverse-J-shaped standardized birth weight specific mortality curve (i.e. a second possible indirect effect of race, not considered by Wilcox-Russell [2,4,5] but equivalent to the interaction of the stressor and birth weight proposed by Hernández-Diaz et al. [6] as a possible alternative cause of the reverse-J shape of birth weight specific infant mortality).

This second indirect effect of race through birth weight (iii) occurs when a change in the variance of birth weight is not reflected in a compensatory change in the shape of the birth weight specific mortality curve. So that the standardized birth weight specific mortality curve changes by race. Finally, the mixing proportion may contribute to the overall observed racial disparities in infant mortality. This is an additional effect of race, which was not discussed in the Wilcox-Russell hypothesis [2,4,5] or its extension by Hernández-Diaz et al. [6]. However, it is similar to the concept of "confounding" in Basso and Wilcox [16,17]. The mixing proportion does involve the birth weight density, nonetheless, the role of birth weight in this case is unclear, depending upon whether birth weight is the cause or the effect of being "compromised." In summary, the CDDmlr provides a reasonable statistical examination of the Wilcox-Russell hypothesis [2,4,5] concerning the potential effects of a stressor, i.e. its direct and/or indirect effects on infant mortality among "normal" as well as "compromised" births [8]. It can potentially distinguish between the "plausible" directed acyclic graphs identified by Hernández-Diaz et al. [6] (Figure 1).

The likelihood function for the basic birth weight (*x*) only CDDmlr model (i.e. CDDmlr without any exogenous covariate) of infant mortality (*y*) is formally defined as a product of the conditional mortality submodel *f*_2_(*y*|*x*; *θ, β*) and the birth weight density submodel *f*_1_(*x*; *θ*):

(1) $$f\left(x,y;\theta ,\beta \right)=f_{2}\left(y|x;\theta ,\beta \right)\cdot f_{1}\left(x;\theta \right)$$

In the case of two truncated Gaussian subpopulations, the birth weight density submodel *f*_1_(*x*; *θ*) is given by

(2) $$f_{1}\left(x;\theta \right)=\pi_{s}\cdot \tilde{N}\left(x;\mu_{s},\sigma_{s}^{2}\right)+\left(1-\pi_{s}\right)\cdot \tilde{N}\left(x;\mu_{p},\sigma_{p}^{2}\right)$$

(3) $$logit\left(\pi_{s}\right)=\eta_{s}$$

*π*_*s*_, the mixing proportion, is defined as the proportion of births belonging to the less numerous of the two subpopulations, that is, the secondary subpopulation (*s*, "compromised" subpopulation) as opposed to the primary subpopulation (*p*, "normal" subpopulation). The reparameterization of *π*_*s *_(Eq. 3) transforms the 0 and 1 bounds on *π*_*s *_to minus and plus infinity, respectively. For *i *= *s *and *p*, $\tilde{N}\left(x;\mu_{i},\sigma_{i}^{2}\right)$ represents the Gaussian density, truncated at 500 grams, with mean *μ_i _*and variance $\sigma_{i}^{2}$. The conditional mortality submodel *f_2_*(*y*|*x*; *θ*, *β*) with two subpopulations is given by

(4) $$f_{2}\left(y|x;\theta ,\beta \right)=q_{s}\left(x;\theta \right)\cdot P_{s}\left(y|x;\beta_{s}\right)+\left[1-q_{s}\left(x;\theta \right)\right]\cdot P_{p}\left(y|x;\beta_{p}\right)$$

where *q_*s*_*(*x*; *θ*) is the probability that an infant with birth weight *x *belongs to the *s *subpopulation. For *i *= *s *and *p*,

(5) $$P_{i}\left(y|x;\beta_{i}\right)=\frac{e^{y\cdot \left[A_{i}+C_{i}\cdot {\left(x-B_{i}\right)}^{2}\right]}}{1+e^{A_{i}+C_{i}\cdot {\left(x-B_{i}\right)}^{2}}}$$

The birth weight density submodel *f*_1_(*x*; *θ*) (Eq. 2) determines that

(6) $$q_{s}\left(x;\theta \right)=\frac{\pi_{s}\cdot \tilde{N}\left(x;\mu_{s},\sigma_{s}^{2}\right)}{\pi_{s}\cdot \tilde{N}\left(x;\mu_{s},\sigma_{s}^{2}\right)+\left(1-\pi_{s}\right)\cdot \tilde{N}\left(x;\mu_{p},\sigma_{p}^{2}\right)}$$

Overall, there are 11 parameters, five defining the birth weight distribution, and six defining the subpopulation-specific mortalities.

In this study, the basic CDDmlr model is extended in two ways. First, we have used European American births as the default and defined the African American "race" effect as an indicator variable (*z*) on each of the 11 parameters in the basic CDDmlr model. Second, for *i *= *s *and *p*, standardized birth weight ($x_{i}^{*}$, i.e. *x *is standardized according to the respective subpopulation mean and standard deviation) is used in the corresponding logistic regression function. Thus

(7) $$f_{1}\left(x|z;\theta \right)=\pi_{s}\left(z\right)\cdot \tilde{N}\left(x;\mu_{s}\left(z\right),\sigma_{s}\left(z\right)\right)+\left[1-\pi_{s}\left(z\right)\right]\cdot \tilde{N}\left(x;\mu_{p}\left(z\right),\sigma_{p}\left(z\right)\right)$$

(8) $$logit\left(\pi_{s}\left(z\right)\right)=\eta_{s}\left(z\right)=\eta_{s,0}+z\cdot \eta_{s,1}$$

(9) $$\mu_{i}\left(z\right)=\mu_{i,0}+z\cdot \mu_{i,1}$$

(10) $$\sigma_{i}\left(z\right)=\sigma_{i,0}+z\cdot \sigma_{i,1}$$

(11) $$P_{i}\left(y|x_{i}^{*},z;\beta_{i}^{*}\right)=\frac{e^{y\cdot \left\{A_{i}^{*}\left(z\right)+C_{i}^{*}\left(z\right)\cdot {\left[x_{i}^{*}-B_{i}^{*}\left(z\right)\right]}^{2}\right\}}}{1+e^{A_{i}^{*}\left(z\right)+C_{i}^{*}\left(z\right)\cdot {\left[x_{i}^{*}-B_{i}^{*}\left(z\right)\right]}^{2}}}$$

(12) $$A_{i}^{*}\left(z\right)=A_{i,0}^{*}+z\cdot A_{i,1}^{*}$$

(13) $$B_{i}^{*}\left(z\right)=B_{i,0}^{*}+z\cdot B_{i,1}^{*}$$

(14) $$C_{i}^{*}\left(z\right)=C_{i,0}^{*}+z\cdot C_{i,1}^{*}$$

This extended model includes 22 parameters, 11 representing the characteristics of European American births, and 11 representing the differences of African compared to European American birth outcomes, that is, the "race" effect. The 5 indicator variable terms in the density submodel (i.e. *η*_1_, *μ_i_*_, 1_, and *σ_i, 1 _*for *i *= *s *and *p*) account for the effects of "race" on the birth weight distribution, while the 6 indicator variable terms in the mortality submodel (i.e. $A_{i,1}^{*}$, $B_{i,1}^{*}$, and $C_{i,1}^{*}$ for *i *= *s *and *p*) account for the racial difference in the standardized birth weight specific mortality curves.

### Model Fitting

The birth weight density and mortality submodels are fitted simultaneously to individual level data using the method of maximum likelihood (ms() in the SPLUS statistical library [20]). The likelihood functions, as defined by Eqs. 7-14, are used except that the 2^nd ^degree polynomial of standardized birth weight specific mortality curves are fitted in linear form, and then transformed to non-linear form after fitting. This significantly reduces the computational resources necessary to fit the model. The resulting parameter estimates are presented in Table 2. This model shows no evidence of lack of fit based on the Hosmer-Lemeshow statistic (with a p-value of 0.66 and 0.41 for females and males, respectively). Bias-adjusted 95% confidence intervals are estimated from 200 bootstrap samples of 200,000 births each, which are randomly generated from the entire birth cohort (as opposed to the more conventional procedure of re-sampling with replacement from the original sample a sample the same size as the original sample). The conventional procedure requires excessive computational resources. An independent study using maternal education as a binary exposure variable suggests that our bootstrap results are consistent with results from the conventional bootstrap method.

**Table 2:** Parameter estimates for the 2001 sample populations

Birth Cohort	z = 0	z = 1	z = 0	z = 1
	**Eur. Am. F**.	**Af. Am. F**.	**Eur. Am. M**.	**Af. Am. M**.
				
*η*_*s, 0*_	-2.75		-2.62	
*η*_*s, 1*_		0.46		0.35
*μ*_*s, 0*_	2678		2739	
*μ*_*s, 1*_		-639		-706
*σ*_*s, 0*_	1098		1098	
*σ*_*s, 1*_		205		238
*μ*_*p, 0*_	3380		3509	
*μ*_*p, 1*_		-211		-221
*σ*_*p, 0*_	455		474	
*σ*_*p, 1*_		0^+^		-2^+^
A*_s, 0_	-7.12		-7.11	
B*_s, 0_	1.06		1.29	
C*_s, 0_	0.77		0.68	
A*_s, 1_		-1.73		-0.83^+^
B*_s, 1_		0.36^+^		0.11^+^
C*_s, 1_		0.54		0.62
A*_p, 0_	-6.85		-6.57	
B*_p, 0_	0.97		1.22	
C*_p, 0_	0.25		0.20	
A*_p, 1_		0.75		0.69
B*_p, 1_		-0.15^+^		-0.44
C*_p, 1_		0.01^+^		0.05^+^

Eur. = European; Af. = African; Am. = American; F. = females; M. = males

^+^: = not significantly different from zero based on the bias-adjusted 95% CI

### Decomposition of the Racial Disparity

Decomposition of the racial disparity is carried out in two steps. First, the total absolute racial disparity in infant mortality is decomposed into deaths attributable to differences in the mixing proportion and rate effects for "normal" and "compromised" births using standard Kitagawa decomposition [21].

The subpopulation specific disparities (rate effects) are further decomposed into direct (independent of birth weight) and indirect (potentially causal through birth weight) effects by factoring the subpopulation relative risks into direct and indirect multiplicative components. The probability of infant death for a European American birth with $x_{i}^{*}$ is given by

(15) $$P_{i}\left(y=1|z=0,x_{i}^{*};\beta_{i}^{*}\right)=\frac{e^{A_{i,0}^{*}+C_{i,0}^{*}\cdot {\left(x_{i}^{*}-B_{i,0}^{*}\right)}^{2}}}{1+e^{A_{i,0}^{*}+C_{i,0}^{*}\cdot {\left(x_{i}^{*}-B_{i,0}^{*}\right)}^{2}}}$$

And the overall infant mortality for the *i *subpopulation of European American births is the weighted average probability of infant death across all birth weights, that is

(16) $$\bar{P_{i}}\left(y=1|z=0;\beta_{i}^{*}\right)=\frac{\sum \left[\tilde{N}\left(x|z=0;\theta \right)\cdot P_{i}\left(y=1|z=0,x_{i}^{*};\beta_{i}^{*}\right)\right]}{\sum \tilde{N}\left(x|z=0;\theta \right)}$$

For an African American birth with $x_{i}^{*}$ in the *i *subpopulation, the probability of death for an African American birth with is given by

(17) $$P_{i}\left(y=1|z=1,x_{i}^{*};\beta_{i}^{*}\right)=\frac{e^{\left(A_{i,0}^{*}+A_{i,1}^{*}\right)+\left(C_{i,0}^{*}+C_{i,1}^{*}\right)\cdot {\left[x_{i}^{*}-\left(B_{i,0}^{*}+B_{i,1}^{*}\right)\right]}^{2}}}{1+e^{\left(A_{i,0}^{*}+A_{i,1}^{*}\right)+\left(C_{i,0}^{*}+C_{i,1}^{*}\right)\cdot {\left[x_{i}^{*}-\left(B_{i,0}^{*}+B_{i,1}^{*}\right)\right]}^{2}}}$$

And the overall infant mortality for the *i *subpopulation of African American births is the weighted average probability of infant death across all birth weights, that is

(18) $$\bar{P_{i}}\left(y=1|z=1;\beta_{i}^{*}\right)=\frac{\sum \left[\tilde{N}\left(x|z=1;\theta \right)\cdot P_{i}\left(y=1|z=1,x_{i}^{*};\beta_{i}^{*}\right)\right]}{\sum \tilde{N}\left(x|z=1;\theta \right)}$$

The overall relative risk of infant death for African American births as compared to European American births in the *i *subpopulation is given by

(19) $$RR_{i}=\frac{\bar{P_{i}}\left(y=1|z=1;\beta_{i}^{*}\right)}{\bar{P_{i}}\left(y=1|z=0;\beta_{i}^{*}\right)}=F_{i1}\cdot F_{i2}$$

(20) $$F_{i,1}=e^{A_{i,1}^{*}}$$

(21) $$F_{i,2}=\frac{\sum {\left[\tilde{N}\left(x_{i}|z=1;\theta \right)\cdot P_{i}\left(y=1|z=1,x_{i}^{*};\beta_{i}^{*}\right)\right]}^{}}{\sum \tilde{N}\left(x_{i}|z=1;\theta \right)\cdot e^{A_{i,1}^{*}}\cdot \bar{P_{i}}\left(y=1|z=0,\beta_{i}^{*}\right)}$$

*F_i_*_,1 _is referred to as the direct factor of "race" in the *i *subpopulation. It is a constant, and independent of birth weight. *F_i_*,2 is referred to as the indirect factor of "race". It represents the combined effect of all birth weight related factors on the racial disparity in infant mortality of the *i *subpopulation. In particular, birth weight related factors include differences in the shape and the horizontal shift of the reverse-J-shaped standardized birth weight specific mortality curve, the non-linear transformation between the probability, and the logit of infant death at any standardized birth weight, as well as the difference in the truncating value of the standardized birth weight distributions between African and European American births.

## Results

### Characteristics of Race Specific Birth Weight Distributions and Infant Mortality

**Figure 2:**
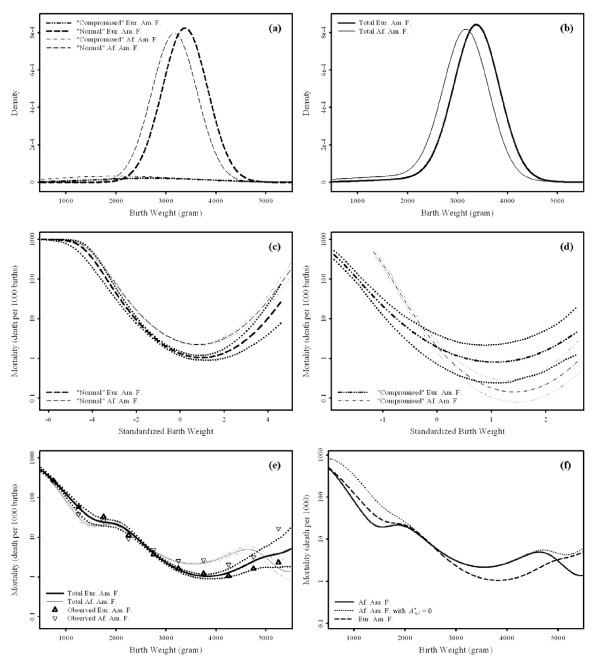
**Model-estimated birth weight distributions and (standardized) birth weight specific infant mortality curves with bias-adjusted 95% confidence intervals for European American females (Eur. Am. F.) and African American females (Af. Am. F.)**. Panel (a) presents the subpopulation specific birth weight densities, while panel (b) shows the total birth weight densities. Panel (c) represents the standardized birth weight specific infant mortality of "normal" births, while panel (d) presents the standardized birth weight specific infant mortality of "compromised" births. Panel (e) shows the total birth weight specific infant mortality. Finally panel (f) compares the total birth weight specific infant mortality with and without ($A_{s,1}^{*}=0$) the direct effect in the "compromised" Af. Am. F. subpopulation and the total mortality curve for Eur. Am. F. is also presented.

**Figure 3:**
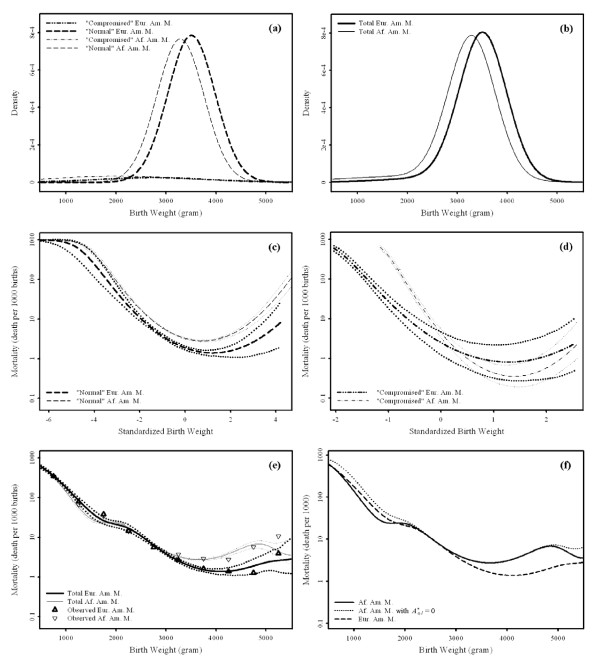
**Model-estimated birth weight distributions and (standardized) birth weight specific infant mortality curves with bias-adjusted 95% confidence intervals for European American males (Eur. Am. M.) and African American males (Af. Am. M.)**. Panel (a) presents the subpopulation specific birth weight densities, while panel (b) shows the total birth weight densities. Panel (c) represents the standardized birth weight specific infant mortality of "normal" births, while panel (d) presents the standardized birth weight specific infant mortality of "compromised" births. Panel (e) shows the total birth weight specific infant mortality. Finally panel (f) compares the total birth weight specific infant mortality with and without ($A_{s,1}^{*}=0$ the direct effect in the "compromised" Af. Am. M. subpopulation and the total mortality curve for Eur. Am. M. is also presented.

The qualitative characteristics of the birth weight distributions and birth weight specific infant mortality are similar for both races (Table 3). The "normal" subpopulation accounts for 90.6-94.0% of births, while the remaining births are classified as "compromised". The "normal" subpopulation has mean birth weight in the normal birth weight range, 3169-3509 grams, and a relatively small standard deviation in birth weight, 455-474 grams. On the other hand, the "compromised" subpopulation has a lower mean birth weight, 2034-2739 grams, and a very large standard deviation in birth weight, 1098-1336 grams. Although it represents less than 10% of births in either race, the "compromised" subpopulation accounts for the majority of low birth weight and macrosomic births (Figures 2a and 3a). Further the "compromised" subpopulation has generally lower birth weight specific infant mortality (Figures 2c-2d, and 3c-3d) but a higher death rate overall (Table 3). This is due to Simpson's paradox, that is, because the "compromised" subpopulation accounts for the majority of low birth weight and macrosomic births, where mortality tends to be higher. Overall, the "normal" subpopulation generally accounts for 49.0-63.1% of total infant deaths, while the "compromised" subpopulation accounts for the remaining deaths (Table 3).

**Table 3:** Model-estimated birth weight distribution and mortality characteristics for the 2001 sample populations

**Birth Cohort**	**Eur. Am. F**.		**Af. Am. F**.		**Eur. Am. M**.		**Af. Am. M.**	
				
	**estimate**	**LCI UCI**	**estimate**	**LCI UCI**	**estimate**	**LCI UCI**	**estimate**	**LCI UCI**
"Normal" Subpopulation								
Proportion (%)	94.0	(93.5; 94.3)	90.7	(90.5; 91.0)	93.2	(92.7; 93.6)	90.6	(90.5; 90.8)
Mean (g)	3380	(3378; 3382)	3169	(3168; 3170)	3509	(3507; 3512)	3288	(3287; 3289)
Standard Deviation (g)	455	(453; 457)	455	(454; 457)	474	(472; 476)	472	(471; 473)
LBW Rate (%)	2.8	(2.7; 2.9)	7.0	(6.9; 7.0)	1.8	(1.7; 1.9)	4.8	(4.7; 4.9)
Death Rate ^#^	2.3	(2.1; 2.6)	4.4	(4.2; 4.5)	2.9	(2.7; 3.2)	5.2	(5.0; 5.4)
Percent of Total DR (%)	63.1	(58.2; 67.8)	54.7	(53.5; 56.1)	60.5	(55.6; 65.0)	49.0	(47.5; 50.5)
"Compromised" Subpopulation								
Proportion (%)	6.0	(5.7; 6.5)	9.3	(9.0; 9.5)	6.8	(6.4; 7.3)	9.4	(9.2; 9.5)
Mean (g)	2678	(2630; 2730)	2039	(1995; 2072)	2739	(2684; 2785)	2034	(1995; 2072)
Standard Deviation (g)	1098	(1067; 1126)	1303	(1289; 1320)	1098	(1076; 1121)	1336	(1323; 1350)
LBW Rate (%)	42.3	(40.4; 43.8)	58.5	(57.7; 59.2)	40.4	(38.7; 42.4)	58.3	(57.5; 59.1)
Death Rate ^#^	21.2	(18.3; 24.9)	35.5	(33.7; 37.1)	26.3	(23.0; 30.0)	52.4	(50.3; 55.0)
Percent of Total DR (%)	36.9	(32.2; 41.8)	45.2	(43.9; 46.5)	39.5	(35.0; 44.4)	51.0	(49.5; 52.5)
Total Population								
LBW Rate (%)	5.2	(5.1; 5.3)	11.8	(11.7; 11.9)	4.5	(4.4; 4.6)	9.8	(9.8; 9.9)
Death Rate ^#^	3.5	(3.2; 3.7)	7.3	(7.1; 7.4)	4.5	(4.3; 4.8)	9.6	(9.4; 9.9)

Eur. = European; Af. = African; Am. = American; F. = females; M. = males

LCI = lower 95% confidence interval; UCI = upper 95% confidence interval; LBW = low birth weight (i.e. <2500 grams); DR = death rate

^#^: death per 1000 births

### Racial Differences in Birth Weight Distributions

Race has substantial effects on the distribution of birth weight (Table 3 Figure 2a-2b, and 3a-3b). For both sexes, the proportion of "normal" births is approximately 3% smaller and the means of both subpopulations are significantly smaller in African American births compared to European American births. The standard deviation of "compromised" African American births is significantly larger compared to European American births. However, there is no difference in the standard deviation of the "normal" subpopulation between African and European American infants of the same sex. Collectively these differences account for the larger low birth weight rates generally observed in African American birth weight distributions (Table 3) [7,22].

### Racial Differences in Infant Mortality

There are substantial racial differences in infant mortality as well (Table 3 Figures 2c-2e, and 3c-3e). The subpopulation specific results show that African American birth weight specific "normal" mortality is larger than European American mortality (Figures 2c and 3c), while African American birth weight specific "compromised" mortality is generally smaller than European birth weight specific mortality (Figures 2d and 3d). Birth weight specific total mortality shows the "pediatric paradox", that is significantly smaller African American mortality at lower birth weights but larger mortality in the larger birth weight range (Figures 2e and 3e). The lower mortality of African Americans at smaller birth weights is accounted for by the lower mortality of "compromised" African American births compared to European American births at the smaller birth weights where the "compromised" subpopulation predominates (Figures 2d and 3d). Similarly the excess mortality of African Americans in the normal birth weight range is accounted for by the larger mortality of African American "normal" births compared to European American "normal" births in the central part of the birth weight range where "normal" births predominate (Figures 2c and 3c).

The estimated racial disparity can be decomposed into a mixing proportion effect and two rate effects (in particular, one for the "normal" subpopulation and the other for the "compromised" subpopulation) by applying Kitagawa decomposition analysis [21] to the model predicted death rates (Table 3). The results are presented in Table 4. All three effects carry absolute risks of substantial magnitude. The mixing proportion effect is due to the difference in the proportion of "normal" to "compromised" births between African and European American births of the same sex. It accounts for 0.8-0.9 death/1000 in the racial disparities of infant mortality (3.8-5.1 death/1000). The remaining disparity is split between the "normal" and "compromised" subpopulations, about equally in males, while the "normal" subpopulation dominates in females. Thus the mixing proportion and the subpopulation rate effects all account for substantial absolute proportions of the overall racial disparity.

**Table 4:** Kitagawa decomposition of the observed racial disparities in infant mortality (death per 1000 births)

**Decomposition**	**Females**		**Males**	
		
	**estimate**	**LCI UCI**	**estimate**	**LCI UCI**
Mixing Proportion Effect	0.81	(0.67; 0.95)	0.90	(0.72; 1.08)
Rate Effect				
"Compromised"	1.09	(0.79; 1.37)	2.11	(1.75; 2.48)
"Normal"	1.90	(1.67; 2.19)	2.08	(1.77; 2.39)
Total Disparity	3.81	(3.53; 4.07)	5.09	(4.81; 5.40)

LCI = lower 95% confidence interval; UCI = upper 95% confidence interval

### Birth Weight and the Racial Disparity

A further decomposition of the subpopulation specific racial disparities into direct (independent of birth weight) and indirect (potentially causal through birth weight) effects based on relative risks is summarised in Table 5. The overall racial disparities are also presented. We used the method of direct standardization of death rates to calculate the infant mortalities and the relative risks. In particular, we used the European American birth cohorts as the reference (standard) population and applied its distribution in estimating the mortalities of both European and African American births. Therefore, the relative risks in this table do not necessarily match the results in Table 3 due to the truncation at 500 grams. This does not affect the "normal" births, because the truncation occurs at about six standard deviations below the mean. But it does affect the "compromised" subpopulations, which have lower means and large variances of birth weight, and thus the total relative risks as well.

**Table 5:** Subpopulation specific racial effect (relative risk) of infant mortality # decomposed into direct and indirect multiplicative factors

**Racial Effect**	**Females**		**Males**	
		
	**estimate**	**LCI UCI**	**estimate**	**LCI UCI**
"Normal" Subpopulation				
Relative Risk	1.89	(1.71; 2.14)	1.77	(1.63; 1.98)
Direct Factor	2.11	(1.87; 2.49)	2.00	(1.71; 2.68)
Indirect Factor	0.89	(0.78; 1.01)*	0.88	(0.71; 1.00)*
"Compromised" Subpopulation				
Relative Risk	5.19	(4.29; 6.16)	5.12	(4.37; 6.08)
Direct Factor	0.18	(0.01; 0.69)	0.43	(0.11; 1.55)*
Indirect Factor	29.38	(13.83; 91.15)	11.80	(4.31; 33.46)
Total Birth Cohort				
Relative Risk	3.10	(2.83; 3.41)	3.09	(2.89; 3.37)

^#^: mortalities are calculated by the method of direct standardization using European American births as the reference (standard) population

LCI = lower 95% confidence interval; UCI = upper 95% confidence interval

*: relative risk or factor is not significantly different from 1 based on the bias-adjusted 95% CI

Among "normal" births, there is a significant direct effect of being African American that contributes to excess mortality in African American births (Table 5). The indirect effect among "normal" births is marginally insignificant in both males and females and tends to **reduce **African American mortality! The direction of this association is surprising given that mean birth weight of "normal" African American births is significantly smaller than that of European Americans (Table 3, Figures 2a and 3a).

Among "compromised" births, on the other hand, the indirect effect is significant and contributes to the excess African American infant mortalities (Table 5). This excess infant mortality is consistent with expectation, that is higher mortality is associated with a significantly lower birth weight (Table 3). In addition, a direct effect among "compromised" African American births **reduces **infant mortality (Table 5). It is significant for females, but not for males. Since the direct and indirect effects tend to compensate for each other the true size of these effects may exceed the absolute effect predicted for each subpopulation.

Overall, a direct effect on the "normal" subpopulation is responsible for the higher infant mortality of African American births in the normal birth weight range (Figures 2e and 3e), while a direct effect on the "compromised" subpopulation is responsible for the lower infant mortality of African American births at lower birth weights (Figures 2e and 3e). As a result, the race "pediatric paradox" (i.e. African Americans have lower mortality at lower birth weights compared to their European American peers), is due to this beneficial direct effect of being an African American "compromised" birth (Figure 2f and 3f). Finally, a large indirect effect occurs in the "compromised" subpopulation (Table 5).

## Discussion

CDDmlr was designed to examine the Wilcox-Russell hypothesis [2,4,5], and its extensions, e.g. Hernández-Diaz et al. [6], and to provide quantitative estimates of the direct effects, which are independent of birth weight and the indirect effects that may operate through birth weight. As described above we have implemented the same assumptions as Wilcox-Russell [2,4,5] and Hernández-Diaz et al. [6]. Nevertheless, application of a quantitative model has some additional limitations over qualitative models, e.g. data quality and quantity, as well as the details of the implementation.

The analyses are based on the public use samples of the NCHS linked birth death files. These have very large sample sizes (Table 1) so there are unlikely to be issues with power. Birth weight is considered to be reliably measured. Mortality estimates may be slightly biased due to problems associated with linking birth and death certificates. However, these are the same data, with the same problems, that most representative analyses of the US are based upon. For our purposes the most troubling defects are that births at <500 grams and LMP gestational ages <20 weeks are not consistently reported by all states [23]. Following many analyses of these data we have truncated the data to avoid this problem. Consequently, we have used Gaussian distributions truncated at 500 grams to match the data.

One technical difficulty in models of this kind is estimating unbiased direct and indirect effects. The qualitative analysis in Hernández-Diaz et al. [6] is based on the assumptions of counterfactuals [24-26]. Here we take an alternative approach, developed from statistical decision theory [18]. In our case, we have modelled the birth weight density as the sum of two Gaussian distributions and the subpopulation specific mortality curves as a 2^nd ^degree polynomial of Z-scored birth weight standardized with respect to these Gaussians. This eliminates the main effects (associations) of race and birth weight and the logistic regressions can then estimate the direct effect of race on infant mortality versus potential interaction effects of race and birth weight on infant mortality. Direct and indirect effects can be estimated using procedures similar to direct standardization [18]. The result is called a "generated direct effect" by Geneletti [18], which is similar to Pearl's "natural direct effect" [24]. Since the "normal" and "compromised" subpopulations are defined as Gaussian distributions, the appropriate distribution is theoretically available for direct standardization. In this regard, truncation of the data at 500 grams creates a significant truncation difference in the standardized birth weight distributions between African and European American "compromised" births. Consequently, the results based on a common reference population (i.e. the European American distribution, Table 5) may be preferred. Identification issues concerning "generated direct effects" are discussed by Geneletti [18].

One advantage of the decision theory approach is that the assumptions concerning the existence of counterfactuals are not necessary. However, like counterfactual methods, the same strong unmeasured covariate assumptions are required. In particular; a) no unmeasured covariates which affect the stressor (race in this case) and the racial disparities in infant mortality, b) no unmeasured confounding of race and birth weight, and c) no unmeasured confounding of birth weight and infant mortality. Assumption a is necessary to estimate total racial disparities, all three are needed to estimate "generated direct effects" [18].

These assumptions may be less of a problem with race than with other variables such as smoking, which have more precise definitions. Race is typically considered to be socially constructed and defined as that collection of variables (some of which may be observable and some of which are currently unobservable) that are associated in some way with reported race. Given this view, all confounders of racial effects on birth weight or infant mortality, are integral parts of the definition of race. This is the assumption generally used when reporting total "racial disparities", such as those presented in Table 1. Of course it is possible to partial out the effects of measured confounders on racial disparities, e.g. the effects of maternal age, but what are left in this case are simply all the unmeasured and unknown effects of race. The results presented above are uncorrected for confounders, and consequently represent the sum total of all direct and indirect effects associated in some way with race. This should be considered when interpreting the results.

Based on the "pediatric paradox", Wilcox has argued that racial disparities may be underestimated due to unmeasured confounding [5,9-11]. Gage has hypothesized that the lower birth weight specific mortality of African compared to European American "compromised" birth cohorts[10] is due to the heavier fetal loss and selection documented among African Americans [27,28]. If this assumption is correct, then differential fetal loss is associated with the direct effect of being African American in the "compromised" subpopulation and with the "pediatric paradox". This interpretation is also consistent with Platt et al.'s finding [29] that the race "birth weight paradox" disappears when observable fetal deaths (total fetal loss is not observable) are included (as well as live births and infant deaths) in the analysis of racial disparities in infant mortality. Should this selection bias be included in the definition of "race" or should differential fetal loss be excluded from the definition of race? The answer depends upon the question, but CDDmlr potentially makes it possible to correct for this "unmeasured" source of confounding.

Model-based adjustment of this effect yields relative risks of 4.2 and 3.6 for African American female and male births, respectively. These are higher than the predicted total relative risks in Table 5, and much higher than the observed relative risk of 2.1 for both sexes derived from Table 1. This adjusted racial disparity needs to be considered with some caution, since it assumes that the direct effect in the "compromised" subpopulation is completely due to selection bias and can be reduced to zero while all other modelled effects remain the same. Nevertheless, it is possible that a substantial part of the racial disparity in infant mortality is hidden by differential fetal loss.

We assume that unmeasured confounding of birth weight and infant mortality (assumption c) is responsible for the reverse-J shape of the birth weight specific mortality curve [16,17] and that the reverse-J shape is not a "causal" effect of birth weight. We have implemented the characteristic reverse-J shape of birth weight specific infant mortality using a second-degree polynomial to account for this unmeasured confounding. This could cause some error if it cannot adequately represent the shape determined by the unmeasured covariates assumed to be responsible for this phenomenon (Figure 1). A 2^nd ^degree polynomial, however, is a relatively flexible function, and is considered to provide an optimal fit to birth weight specific mortality in the homogeneous case [30].

Moreover, the CDDmlr model corrects for some unmeasured confounding of birth weight and infant mortality, referred to as "normal" versus "compromised" births. It is unlikely that dividing birth cohorts into two Gaussian subpopulations will account for all of the unmeasured confounding between birth weight and infant mortality. Nevertheless, the two subpopulations display significantly different mortality patterns indicating that the CDDmlr model accounts for some otherwise unmeasured heterogeneity [9,10]. In particular, we have argued that the generally higher "normal" birth weight specific mortality compared to "compromised" birth weight specific mortality is due to greater fetal loss among "compromised" births, resulting in a highly selected "compromised" sample at live birth [9,10] similar to the hypothesis concerning the "pediatric paradox". If correct, this effect would violate assumption c, unless the two subpopulations are examined separately, as they are here.

The statistical results presented above (Tables 2 and 5) are consistent with the Wilcox-Russell hypothesis [2,4,5], and its extensions [6] (Figure 1c) that suggest that birth weight is not on the "causal pathway" to infant mortality at least for "normal" births. The racial disparity in birth weight has no significant association with the racial disparity in infant mortality after controlling for the other paths in Figure 1c. There is no evidence of any residual difference in infant mortality between birth weight and infant mortality over and above the direct effect and the reverse-J shape of the standard population, European American births in this case. It is unlikely that this result is compromised by uncontrolled confounding of birth weight and infant mortality, since this would require that the sum total of associations generated by uncontrolled confounding equal zero. It is more likely that all of the effects of race on infant mortality in this subpopulation operate through pathways that do not include birth weight.

On the other hand, there is a substantial indirect effect, which disadvantages African American infant mortality among "compromised" births (Table 5). The results in Table 2 indicate that this association is largely due to a change in shape of the reverse-J-shaped birth weight specific mortality curve between the races. This could be due to an interaction of race and birth weight on infant mortality, or due to a violation of no unmeasured confounding assumptions b or c. It is also equivalent to the interaction [6] required by Figure 1a and also possible in Figure 1b, both of which require that birth weight be on the "causal pathway" to infant mortality. In any event an association between birth weight and infant mortality can not be excluded, and it remains possible that birth weight has a "causal" effect on infant mortality among these "compromised" births.

Overall, the findings suggest that interventions with respect to birth weight will not reduce racial disparities in mortality among "normal" births, but might reduce them among "compromised" births. Identification of the exact mechanisms and whether birth weight plays a "causal" role conditional on "compromised" birth will require additional analysis, i.e. control of potential confounding. The "compromised" subpopulation accounts for about 29-41% of the observed racial disparity for females and males respectively (Table 4).

If our hypothesis concerning the selection effects of fetal loss on observed racial disparities is correct, then the total racial disparity is higher than observed, and the proportion of the disparity due to the "compromised" subpopulation is larger than observed. The confounding, represented by the mixing proportion, accounts for an additional 17-21% of the observed racial disparity for males and females, respectively (Table 4). Nevertheless, completely eliminating the "compromised" subpopulation would a) reduce both the low and the macrosomic birth weight rates, which are generally associated with elevated infant mortality in both African and European American birth cohorts, b) reduce the size of the racial disparity if direct standardization based on the European American distribution are accepted, c) reduce the size of the disparity yet again if our hypothesis concerning the selection effects of fetal loss in the "compromised" subpopulation is correct and included as a potential bias, but d) still result in a population with a racial disparity of 1.9 and 1.8 for females and males, respectively (Table 5), about the level of the relative risk currently observed in the raw data (2.1 for both sexes, Table 1).

## Conclusions

Our results support the Wilcox-Russell [2,4,5] and Hernández-Diaz et al. [6] arguments that birth weight is not on the causal pathway to infant mortality at least among "normal" births. Improvements in birth weight may not necessarily impact infant mortality for these births! However, birth weight cannot be eliminated as a potential cause of infant mortality among a small subpopulation of "compromised" births, generally accounting for less than 10% of the birth cohort. Improvements in birth weight may reduce infant mortality among certain births.

The true racial disparity in infant mortality between African and European American birth cohorts may be obscured by unobserved heterogeneity. This heterogeneity may be due to differential fetal loss, which appears to account for the "pediatric paradox". The true racial disparities may also be obscured by lack of consistently reporting births at below 500 grams in the NCHS linked birth death files.

Part of the racial disparity is due to mixing proportion effects, i.e. a larger number of "compromised" births among African Americans than European Americans. Reducing the disparity in the size of "compromised" births will somewhat reduce racial disparities. If all "compromised" births could be eliminated (i.e. eliminating all possible statistically significant birth weight dependent effects), the racial disparities would decrease slightly (1.9 and 1.8 for females and males, respectively) from the currently observed level (2.1 for both sexes). Therefore, the complete elimination of racial disparities in infant mortality requires the elimination of birth weight independent (i.e. direct) effects, as well as any birth weight dependent (i.e. indirect) effects.

## Competing interests

The authors declare that they have no competing interests.

## Authors' contributions

TBG planned the analysis and wrote the manuscript. FF and EKO carried out the analysis and helped revise the manuscript. AGD consulted on statistical matters and also helped revise the manuscript. All authors have read and approved this manuscript.

## Pre-publication history

The pre-publication history for this paper can be accessed here:

http://www.biomedcentral.com/1471-2393/10/86/prepub

## References

[B1] Mosely WH Chen LC Mosely WH, Chen LC An analytical framework for the study of child survival in developing countries Population and Development Review Supplement to 1984 10 Cambridge: Cambridge University Press

[B2] Wilcox AJ On the importance - and the unimportance - of birthweight Int J Epidemiol 2001 30 6 1233 1241 10.1093/ije/30.6.1233 11821313

[B3] Wise PH The anatomy of a disparity in infant mortality Annu Rev Public Health 2003 24 341 362 10.1146/annurev.publhealth.24.100901.140816 12471271

[B4] The Analysis of Birth Weight and Infant Mortality: an Alternative Hypothesis http://eb.niehs.nih.gov/bwt/index.htm

[B5] Wilcox AJ Russell I Why small black infants have a lower mortality-rate than small white infants - the case for population-specific standards for birth-weight J Pediatr 1990 116 1 7 10 10.1016/S0022-3476(05)81638-5 2295966

[B6] Hernández-Diaz S WA Schisterman EF Hernán MA From causal diagrams to birth weight-specific curves of infant mortality 2008 23 3 163 166 10.1007/s10654-007-9220-4 PMC2722961 18224448

[B7] US-DOHHS Healthy People 2010: Understanding and improving health 2000 Washington, DC: US Department of Health and Human Services 91 95

[B8] Gage TB Fang F O'Neill E Stratton H Maternal age and infant mortality: a test of the Wilcox-Russell hypothesis Am J Epidemiol 2009 169 3 294 303 10.1093/aje/kwn308 19029004 PMC2638945

[B9] Gage TB Birth-weight-specific infant and neonatal mortality: effects of heterogeneity in the birth cohort Hum Biol 2002 74 2 165 184 10.1353/hub.2002.0020 12030647

[B10] Gage TB Bauer MJ Heffner N Stratton H Pediatric paradox: heterogeneity in the birth cohort Hum Biol 2004 76 3 327 342 10.1353/hub.2004.0045 15481671

[B11] Wilcox AJ Infant-mortality among blacks and whites N Engl J Med 1992 327 17 1243 1243 10.1056/NEJM199210223271714 1406810

[B12] Gage TB Therriault G Variability of birth-weight distributions by sex and ethnicity: Analysis using mixture models Hum Biol 1998 70 3 517 534 9599943

[B13] Gage TB Classification of births by birth weight and gestational age: An application of multivariate mixture models Ann Hum Bio 2003 30 5 589 604 10.1080/03014460310001592678 12959900

[B14] Cassady G Strange M The small-for-gestational-age (SGA) infant 1987 Philadelphia: J. B. Lippincott

[B15] Godfrey KM Barker DJP Fetal nutrition and adult disease Am J Clin Nutr 2000 71 5 1344 1352 10.1093/ajcn/71.5.1344s 10799412

[B16] Basso O Wilcox AJ Weinberg CR Birth weight and mortality: causality or confounding? Am J Epidemiol 2006 164 4 303 311 10.1093/aje/kwj237 16847040

[B17] Basso O Wilcox AJ Intersecting birth weight-specific mortality curves: solving the riddle Am J Epidemiol 2009 169 7 787 797 10.1093/aje/kwp024 19240224 PMC2727223

[B18] Geneletti S Identifying direct and indirect effects in a non-counterfactual framework J R Stat Soc Ser B 2007 69 2 199 215 10.1111/j.1467-9868.2007.00584.x

[B19] Schisterman EF Whitcomb BW Mumford SL Platt RW Z-scores and the birthweight paradox Paediatr Perinat Epidemiol 2009 23 5 403 413 10.1111/j.1365-3016.2009.01054.x 19689489 PMC2742985

[B20] Bates DM Chambers JM Chambers JM, Hastie TJ Nonlinear models Statistical Models in S 1992 Pacific Grove, CA: Wadsworth and Brooks 421 454

[B21] Gupta PD A general method of decomposing a difference between two rates into several components 1978 15 1 99 112 631402

[B22] Sappenfield WM Buehler JW Binkin NJ Hogue CJR Strauss LT Smith JC Differences in neonatal and postneonatal mortality by race, birth weight, and gestational age Public Health Rep 1987 102 2 182 192 3104975 PMC1477813

[B23] US-DOHHS State Definitions and Reporting Requirements for Live Births, Fetal Deaths, and Induced Terminations of Pregnancy 1997 Washington, DC: US Department of Health and Human Services

[B24] Pearl J Causal inference in statistics: an overview Stat Surv 2009 3 96 146 10.1214/09-SS057

[B25] VanderWeele TJ Marginal structural models for the estimation of direct and indirect effects Epidemiology 2009 20 1 18 26 10.1097/EDE.0b013e31818f69ce 19234398

[B26] Robins JM Hernán M Brumback B Marginal structural models and causal inference in epidemiology Epidemiology 2000 11 5 550 560 10.1097/00001648-200009000-00011 10955408

[B27] Buck GM Shelton JA Mahoney MC Michalek AM Powell EJ Racial variation in spontaneous fetal deaths at 20 weeks or older in upstate New York, 1980-86 Public Health Rep 1995 110 5 587 592 7480613 PMC1381636

[B28] Kallan JE Race, intervening variables, and 2 components of low-birth-weight Demography 1993 30 3 489 506 10.2307/2061653 8405611

[B29] Platt RW Joseph KS Ananth CV Grondines J Abrahamowicz M Kramer MS A proportional hazards model with time-dependent covariates and time-varying effects for analysis of fetal and infant death Am J Epidemiol 2004 160 3 199 206 10.1093/aje/kwh201 15257989

[B30] Fryer JG Hunt RG Simons AM Falkner F Biostatistical considerations: the case for using models Prevention of Perinatal Mortality and Morbidity 1984 Basel, Switzerland: Karger 9 30

